# Optimal methods for fitting probability distributions to propagule retention time in studies of zoochorous dispersal

**DOI:** 10.1186/s12898-016-0057-0

**Published:** 2016-02-01

**Authors:** Duarte S. Viana, Luis Santamaría, Jordi Figuerola

**Affiliations:** Estación Biológica de Doñana (EBD-CSIC), C/Américo Vespucio, s/n, 41092 Seville, Spain; CIBER Epidemiología y Salud Pública (CIBERESP), Seville, Spain

**Keywords:** Seed dispersal, Dispersal kernel, Probability distribution, Endozoochory, Epizoochory, Gut passage time

## Abstract

**Background:**

Propagule retention time is a key factor in determining propagule dispersal distance and the shape of “seed shadows”. Propagules dispersed by animal vectors are either ingested and retained in the gut until defecation or attached externally to the body until detachment. Retention time is a continuous variable, but it is commonly measured at discrete time points, according to pre-established sampling time-intervals. Although parametric continuous distributions have been widely fitted to these interval-censored data, the performance of different fitting methods has not been evaluated. To investigate the performance of five different fitting methods, we fitted parametric probability distributions to typical discretized retention-time data with known distribution using as data-points either the lower, mid or upper bounds of sampling intervals, as well as the cumulative distribution of observed values (using either maximum likelihood or non-linear least squares for parameter estimation); then compared the estimated and original distributions to assess the accuracy of each method. We also assessed the robustness of these methods to variations in the sampling procedure (sample size and length of sampling time-intervals).

**Results:**

Fittings to the cumulative distribution performed better for all types of parametric distributions (lognormal, gamma and Weibull distributions) and were more robust to variations in sample size and sampling time-intervals. These estimated distributions had negligible deviations of up to 0.045 in cumulative probability of retention times (according to the Kolmogorov–Smirnov statistic) in relation to original distributions from which propagule retention time was simulated, supporting the overall accuracy of this fitting method. In contrast, fitting the sampling-interval bounds resulted in greater deviations that ranged from 0.058 to 0.273 in cumulative probability of retention times, which may introduce considerable biases in parameter estimates.

**Conclusions:**

We recommend the use of cumulative probability to fit parametric probability distributions to propagule retention time, specifically using maximum likelihood for parameter estimation. Furthermore, the experimental design for an optimal characterization of unimodal propagule retention time should contemplate at least 500 recovered propagules and sampling time-intervals not larger than the time peak of propagule retrieval, except in the tail of the distribution where broader sampling time-intervals may also produce accurate fits.

## Background

The probability distribution of biological variables is of great importance for modeling and understanding biological phenomena, including the mechanistic basis of ecological processes. Mechanistic models are widely used in seed dispersal ecology (used here as a general term for the dispersal ecology of dormant propagules, including spores, resting eggs and cysts of plants, animals and fungi), as propagule movement is often difficult to track [1–5].

A considerable part of the Earth’s biota does not actively move. Instead, they produce dormant propagules that rely on several types of vectors for their dispersal, such as wind, water and animals [3, 6–10]. Among the various vectors, animals such as birds and mammals disperse a great variety of propagules belonging to different species [8, 10–12]. Propagules are dispersed either externally, entangled in the fur or feathers (“epizoochory” hereafter), or internally, following ingestion and while transiting through the animal’s gut (“endozoochory” hereafter).

A key element in the study of animal-mediated dispersal is the estimation of the distance at which propagules are dispersed. Dispersal distance (D) is usually estimated as the product of the vector movement rate (V) and the retention time (R) of ingested or attached propagules (D = V × R). The distribution of dispersal distances, i.e. the dispersal kernel, is a major determinant of the spatial distribution of individuals, populations and species, thus its accurate estimation is of vital importance for studying and modeling metapopulation and metacommunity dynamics [8, 9, 13], as well as the distributions and expansion rates of species [3, 14, 15]. For example, many species distribution models (SDMs), which are used to model how species are distributed along niche gradients, incorporate dispersal kernels to predict range expansions or shifts under different scenarios of environmental change and estimate realistic distributions according to the species’ dispersal potential [16, 17].

Together with the vector’s movement behaviour, propagule retention time has been found to critically affect several properties of propagule dispersal kernels such as the range and frequency of dispersal events, thus the probability of long distance dispersal [4, 18]. Therefore, the accurate characterization of retention times is of fundamental importance for avoiding the magnification of biases already introduced by assumptions about vector movement when estimating propagule dispersal kernels. This is the reason why numerous empirical studies have investigated the different factors affecting propagule retention time, such as the size and shape of plant propagules [19, 20], the developmental stage of animal propagules [21], or the morphology [4, 22, 23], digestive physiology [24, 25], and activity [26, 27] of animal vectors.

However, obtaining continuous measurements of propagule retention time is, in most cases, extremely difficult owing to the ample time span and variable grain required (from minutes to several days, depending on the animal vector and propagule type), as well as monitoring interferences on the animal vector. In endozoochory studies, the most common strategy to measure retention time is to force-feed captive animals and collect their droppings at given time intervals, often of varying length [e.g., 24, 28]. Similarly, the usual practice in epizoochory studies is to record propagule attachment time at given time intervals, by measuring the number of propagules that remain attached to the fur of captive or semi-captive animals [29, 30], as well as to experimental coats [31], from a sample of propagules placed there by hand at the beginning of the experiment. In both cases, propagule retention time (i.e., defecation or detachment time) is recorded as a frequency at the end of given time intervals, thus as a series of interval-censored data.

Nevertheless, the censored nature of these data is usually not taken into account in studies of animal-mediated propagule dispersal (but see [4]). Although this systematic uncertainty on the precise moment of propagule deposition can severely bias the estimation of dispersal distance, fitting procedures used to characterize the distribution of retention times usually assign the frequency of retrieval to the collection time (i.e., to the upper bound of the time interval). Moreover, in most cases the fitting method is not accurately reported or insufficiently described [e.g. 5, 18, 32–36]. We compared the accuracy and robustness of different methods in fitting continuous probability distributions to propagule retention-time data. Because propagule retention-time data typically show right-skewed distributions with an initial peak (corresponding to the distribution mode) followed by a steep decrease and a long tail, we considered three parametric distributions commonly used to characterize these data: the lognormal, gamma and Weibull distributions. We assessed the performance of five different fitting methods. In the first three methods, we fitted parametric probability distributions to empirical distributions using either the (i) lower, (ii) mid or (iii) upper bounds of the sampling intervals as the data points; and in the other two methods, we fitted cumulative parametric distributions to (upper-bound) data arranged as empirical cumulative distributions, using two different procedures: (iv) maximum likelihood (CD-ML) and (v) non-linear least squares (CD-NLS). To assess the performance of these different methods, we applied them to a simulated dataset (based on empirical distributions; see “Methods” section) and compared the resulting parameter estimates and functions to the original ones. In addition, we assessed the robustness of the five fitting methods to variation in the distribution type (lognormal, gamma and Weibull) or parameter values of the original distribution (from which the simulated dataset was sampled), in the sample size (i.e., number of uptake propagules) and in the length of sampling time-intervals used to generate the simulated dataset.

## Results

### Variation in probability distribution

**Fig. 1:**
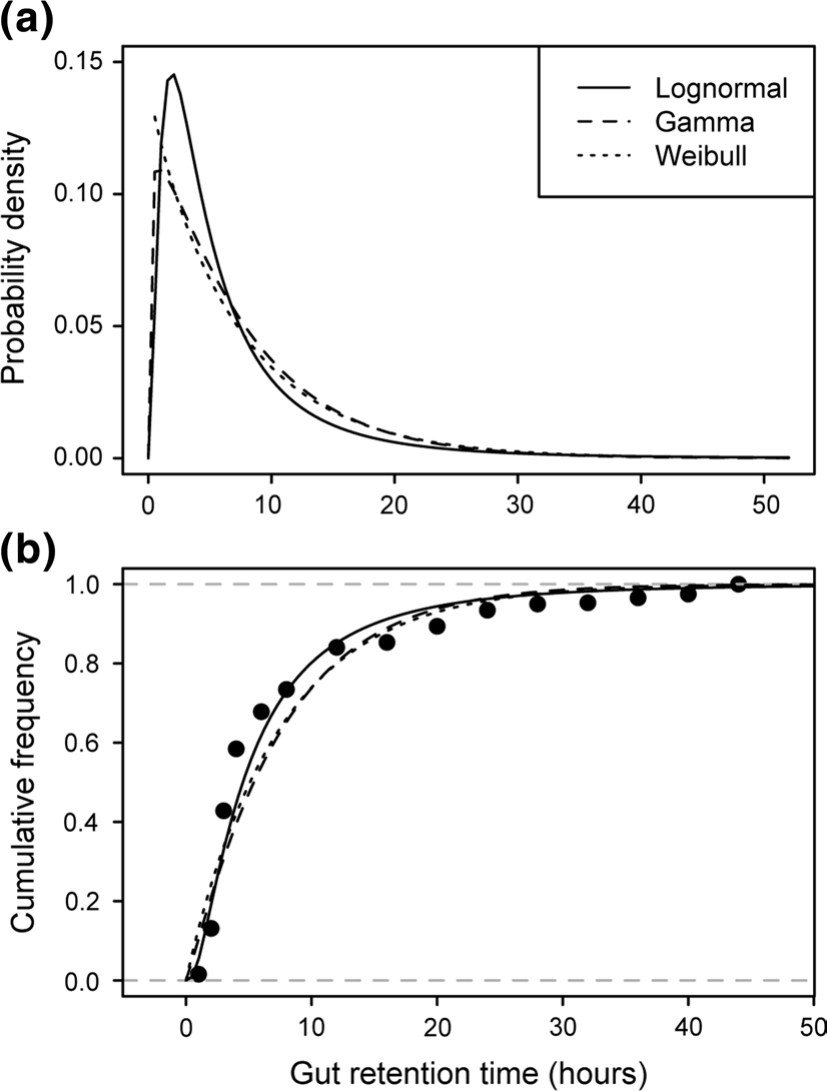
Examples of lognormal, gamma and Weibull distributions fitted to gut retention time of propagules ingested by waterfowl **a** and how these parametric distributions fit to empirical data **b**. Data is taken from [4]

**Fig. 2:**
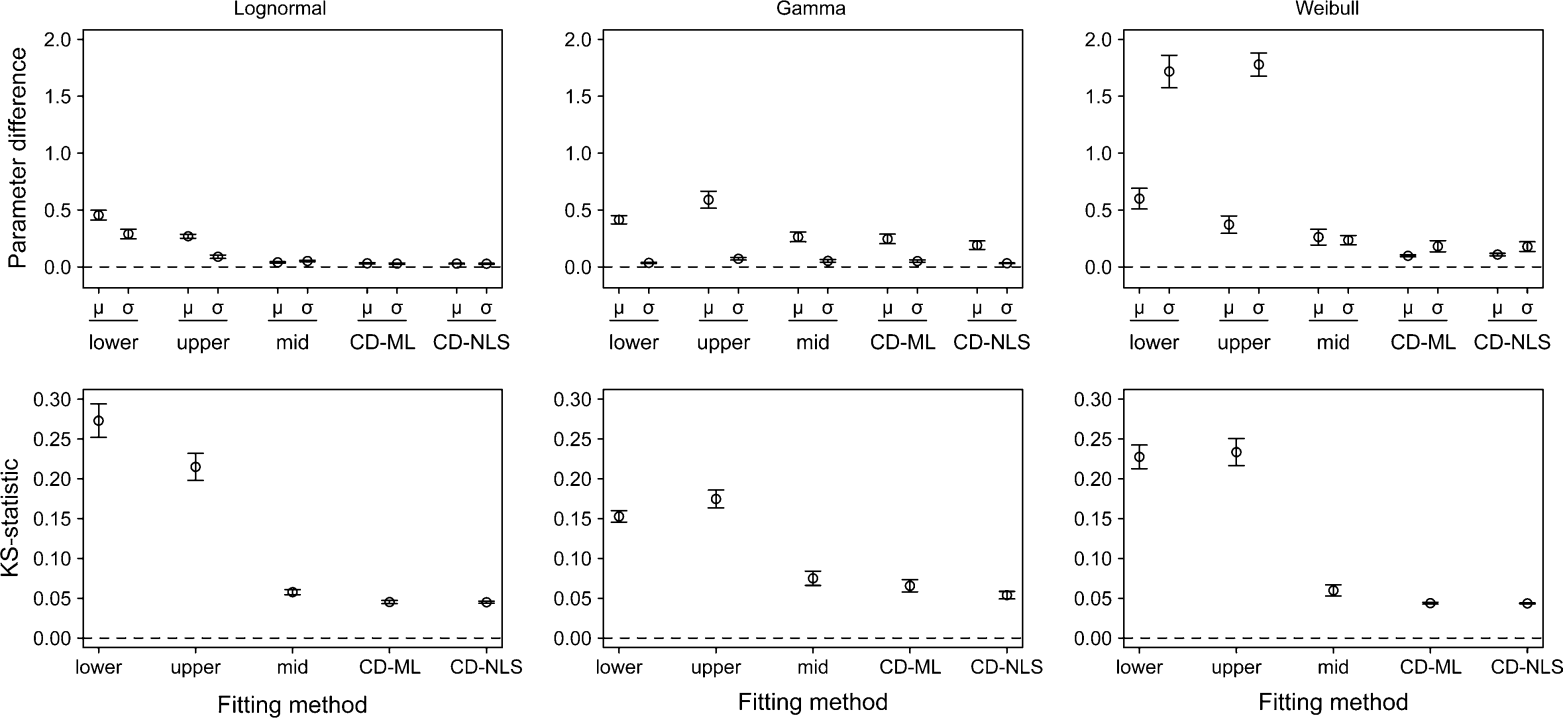
Fitting performance of the different methods to lognormal (*left* panels), gamma (*middle* panels) and Weibull (*right* panels) distributions measured by the difference between the original and fitted parameters (mean ± se; *upper* panels) and the KS-statistic (mean ± se; *lower* panels)

All three types of probability distributions (lognormal, gamma and Weibull) could accurately characterize the distribution of propagule retention times, as exemplified in Fig. 1. Among the different fitting methods, fittings to cumulative distributions (both CD-ML and CD-NLS) provided the most accurate fits for all three types of probability distribution, both in parameter estimates and in the shape of the distribution (KS-statistic; Fig. 2). KS values supported the high accuracy of the estimates obtained with these two methods (KS-statistic <0.05). For fits to interval bounds, those using the upper and lower bounds had worse performances than that using the interval mid-value. The distribution type did affect, however, the accuracy of the different parameter estimates: the location parameter (μ) was less accurately estimated (greater difference between estimated and original parameter values) than the variance parameter (σ) for the lognormal and gamma distributions, while the opposite was true for the Weibull distribution (Fig. 2, upper panels).

### Variation in sample size

**Fig. 3:**
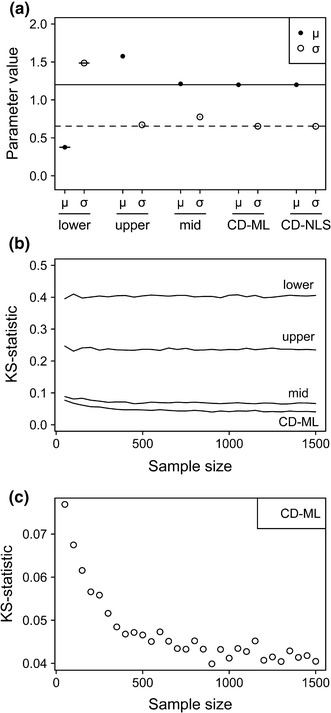
Robustness of the different fitting methods to variation in sample size (ranging from 50 to 1500 propagules). Fitting results correspond to the lognormal distribution. **a** Estimated parameter values (mean ± se). The *solid* and *dashed lines* indicate the original values of the location (μ) and variance (σ) parameters, respectively. Where error bars are undistinguishable, it means that standard errors are smaller than the mean-value dots. **b** KS statistic. **c** Fitting performance of the CD-ML method for different sample sizes, estimated by the KS statistic

Fittings to the cumulative distribution (CD-ML and CD-NLS) were also the most robust against variation in sample size, i.e., variation in the number of retrieved propagules (Fig. 3a, b). Parameters estimated with these two estimation methods were remarkably accurate (Fig. 3a). Despite the low variation in accuracy of CD fits (KS-statistic ranged from 0.04 to 0.08; Fig. 3c), detailed inspection revealed that increasing sample sizes resulted in more accurate CD-ML fits up to N = 500, from which the fitting accuracy levelled off (i.e., reached an asymptotic KS-statistic; Fig. 3c).

### Variation in sampling interval

**Fig. 4:**
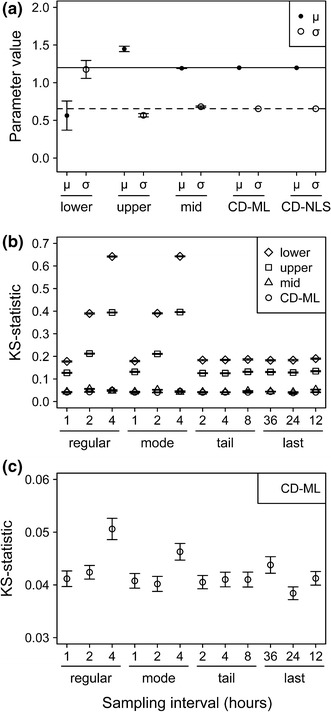
Robustness of the different fitting methods to variation in sampling time-interval. Fitting results correspond to the lognormal distribution. **a** Estimated parameter values (mean ± se). The *solid* and *dashed* lines indicate the original values of the location (μ) and variance (σ) parameters, respectively. Where error bars are undistinguishable, it means that standard errors are smaller than the mean-value dots. **b** KS statistic (mean ± se). **c** Fitting performance of the CD-ML method for different lengths in sampling time-intervals, estimated by the KS statistic. Time-intervals varied in a regular way over the whole sampling period (1, 2 or 4 h up to 52 h), around the distribution mode (1, 2 or 4 h during the first 8 h and 4 h afterwards), in the tail of the distribution (1 h up to 8 h and 2, 4 or 8 h afterwards), and the last time-interval (stopping sampling at 36, 24 or 12 h after propagule ingestion up to 52 h, the time of the last sampling)

Consistently with the previous results, fittings to cumulative distributions (CD-ML and CD-NLS) provided the most accurate parameter estimates and were the most robust against variations in the length of sampling intervals (Fig. 4a, b). Despite the low variation in performance provided by CD fits, a detailed inspection showed that increasing the length of the initial sampling-time intervals (nearby the distribution’s mode) resulted in reduced estimation accuracy, i.e., in increased KS statistics (Fig. 4c).

### Impact of fitting method on estimates of propagule dispersal kernels

Non-optimal methods used to fit propagule retention time distributions produced severe biases in estimated dispersal kernels (Table 1; difference percentages ranged from 0.1 to 123 %). These biases were strongest for the frequency of long distance dispersal: when using the most common method (fits to upper-bound values), it increased by 123 and 19 % for *Potamogeton pectinatus* and *Scirpus lacustris* seeds, respectively. The magnitude of the bias was also related to the sampling protocol, specifically to the sampling time-interval. Experiments using shorter time-intervals around the distribution mode incurred in smaller biases (0.1–19 % in [37], using *S. lacustris* seeds) than those using more spaced intervals (2.4–123 % in [24], using *P. pectinatus* seeds). Overall, kernel properties related to long distance dispersal (namely dispersal over 100 km and the 99th distance percentile) were the most affected, whereas central tendency measures (i.e., mean and median) were less affected by suboptimal fitting methods.

**Table 1:** Biases introduced by the choice of methods used to fit propagule retention time, on the dispersal kernels of two plant species (*Potamogeton pectinatus* Pp, and *Scirpus lacustris* Sl) dispersed by the same vector species (mallard *Anas platyrhyncos*)

Method	Seed sp.	% LDD	Mean (km)	Median (km)	Q99 (km)
CD-ML	Pp	0.15	38.3	18.1	326.3
	Sl	0.47	58.7	20.5	498.5
Lower	Pp	0.13 (−12.0)	48.2 (+25.8)	18.5 (+2.4)	435.5 (+33.4)
	Sl	0.40 (−14.0)	58.6 (−0.3)	20.3 (−0.9)	503.7 (+1.0)
Upper	Pp	0.33 (+123.1)	40.0 (+4.5)	19.0 (+4.7)	303.5 (−7.0)
	Sl	0.55 (+19.1)	61.3 (+4.5)	20.8 (+1.5)	506.1 (+1.5)
Mid	Pp	0.12 (−18.9)	32.4 (−15.3)	17.5 (−3.2)	256.0 (−21.6)
	Sl	0.47 (+0.8)	58.8 (+0.2)	20.5 (+0.1)	499.1 (+0.1)

The comparison is based on four different parameters of the dispersal kernels for which the respective values are given: long-distance dispersal frequency (%LDD; i.e., % of dispersal events with distance >100 km), mean and median distance, and 99th distance percentile (Q99). Values between brackets indicate the magnitude of the bias, i.e., the relative difference (in percentage) between the value obtained using the optimal method (CD-ML) and each of all other fitting methods. Note that the CD-NLS method led to overall similar results to those of CD-ML (but see “Discussion” section)

## Discussion

The use of fitting methods that take into account the interval-censored nature of propagule retention time data proved necessary for a correct estimation of the underlying probability distributions. If we take the example of the lognormal fit to interval upper-bound data, which is the most used fitting method, a difference of 0.27 in the location parameter μ (i.e., 1.3 h in median retention time) was observed in relation to the original parameter. If the vector flies at an average speed of 60 km/h, a common speed for waterfowl species [38], the difference in median dispersal distance (= 1.3 h × 60 km/h) would be 78 km, provided that the vector moves linearly until propagule retrieval. Even if actual vector movement distances are incorporated into dispersal distance estimations, considerable biases are also observed, mostly in the estimation of long distance dispersal (Table 1). The magnitude of these biases stress the necessity of using fitting methods that are able to account for the censored nature of propagule retention time data.

The two estimation methods based on cumulative distributions (CD-ML and CD-NLS) produced accurate estimations for lognormal, gamma and Weibull distributions and were remarkably robust against variations in data quality (sample size and sampling-time interval). The CD-NLS method requires more data points than the CD-ML method to be equally robust, as its estimation did not converge for distributions with a low variance (i.e., resulting from either a reduced retention time range or too large sampling time-intervals). We therefore recommend the CD-ML method, which fits the parameters to the censored data by maximum likelihood, as a general approach to characterize the probability distribution of propagule retention time. It can be implemented via the R package *fitdistrplus* [39] and other software packages such as MATLAB and SAS (this is not an exhaustive list).

The accuracy of estimations with the best method (CD-ML) was high enough to ensure very low deviations from the original distribution, as the maximum observed deviation in cumulative probability (compared to the original probability distribution) was only 0.05 (KS-statistic). Although distribution fittings using the interval mid-points also provided satisfactory results, estimated parameters were not as accurate as those using cumulative distributions. In particular, the variance parameter (σ) was generally overestimated, mostly at low sample sizes. The overestimation of this parameter might result in overestimated dispersal distances and, consequently, overestimated frequencies of long distance dispersal (as inferred from [4, 18]).

The robustness of the CD-ML method suggests that many hitherto obtained datasets on propagule retention time might be properly used in mechanistic models of propagule dispersal, even if the tail of the distribution is undersampled [e.g., 19]). Our results also suggest that the experimental conditions may and should be designed to optimize the accurate characterization of retention-time probability distributions by taking into account the tradeoff between sampling effort and measurement precision. Simulations suggested that (i) at least 500 propagules should be retrieved to obtain more reliable estimates of retention time, and (ii) sampling effort should ensure an accurate characterization of the time peak (i.e., mode) of propagule retrieval by choosing sampling time-intervals of shorter length than the peak retrieval. Although the sampling effort for the distribution tail can be more relaxed, adequate sampling time-intervals should also be used until the end.

## Conclusions

Based on our comparative analysis, we recommend the use of the CD-ML method to fit parametric probability distributions to propagule retention-time data. Because propagule retention time is a key parameter in mechanistic models of passive dispersal [4, 18], an accurate parametric characterization of its probability distribution contributes to more reliable estimations of dispersal kernels and shadows. Given that many plants, invertebrates and microbes (including aquatic taxa) rely on passive dispersal, and that dispersal is a key determinant of biodiversity distribution patterns, the methodology presented in this study will also be useful for modeling population and community dynamics (e.g., meta-population and -community models), as well as species distributions (e.g., species distribution models; SDMs).

## Methods

We assessed the performance of the five different procedures used to fit parametric probability distributions to “empirical” datasets by comparing the distributions obtained from such fits with the original distributions from which the “empirical” datasets were randomly sampled. Original distributions were aimed at representing the propagule retention time of a given vector species; hence, we obtained them from a study in which the gut retention time of seeds fed to several waterfowl species was measured and fitted to three types of probability distribution—lognormal, gamma and Weibull (Fig. 1) [4]. These distributions are suited to characterize the distribution of propagule retention times, and although having a reduced number of parameters, their flexibility allows them to represent a broad variety of curve shapes [4, 33].

The general procedure was as follows. First, we generated the “empirical dataset” by drawing a random sample of the original distribution of propagule retention times and assigning the resulting values to predefined sampling time-intervals (thus simulating empirical sampling in real-world studies). We repeated this procedure 100 times for each original probability distribution to account for random variation. Second, we fitted a probability distribution (of the same type as the original one) to the empirical dataset, using the five procedures outlined above—i.e., fits to either the lower-bound, mid-interval or upper-bound values of the corresponding time interval, or fits to the cumulative distribution of empirical, upper-bound data. In the first three methods, interval bounds (either lower, mid or upper points) were considered to represent a continuous variable and the parameters of the fitted distributions were estimated by maximum likelihood (i.e., calculated according to probability density). For the methods using the empirical cumulative distribution, we estimated the parameters of fitted distributions either by maximum likelihood (method hereafter termed CD-ML), according to the procedure presented in Delignette-Muller and Dutang [40] (see [41, 42] for further details), or by non-linear least squares (method hereafter termed CD-NLS). All fittings were performed in R [43] using package *fitdistrplus* [39] for maximum likelihood estimation (function *fitdist* for the fittings using the time-interval bounds, and function *fitdistcens* for the CD-ML method), and the R base package for the CD-NLS method (function *nls*). We then assessed the fitting performance by comparing estimated and original distribution parameters (difference in value), and by estimating the Kolmogorov–Smirnov (KS) statistic using the package *kolmin* [44] in R [43]. The KS-statistic was obtained by drawing a random sample of the fitted distribution (N = 500) and comparing it with the original distribution. It represents the maximum difference in cumulative probability between the reference and focal distributions, thus corresponding to a goodness-of-fit measure ranging from zero (i.e., 100 % accurate) to one.

### Robustness of the different fitting methods

We assessed the robustness of the five fitting methods by simulating natural variation in propagule retention time (i.e., by varying the type and shape of original distributions) and different experimental designs commonly found in the literature, namely variation in sample size and in the length of sampling-time intervals. We applied the procedures described above to the sets of simulated data described below.

#### Variation in distribution type and shape

We assessed the robustness of the fitting method to variation in the probability distribution by using three distribution types (lognormal, gamma and Weibull) and 30 different sets of parameters (variation in parameter values) to generate the original distributions from which “empirical datasets” were randomly drawn. To obtain a representative set of parameter combinations for each distribution type, we applied a Latin Hypercube Sampling procedure to the range of parameter variation reported in Viana et al. [4], using the R package *lhs* [45].

#### Variation in sample size

We assessed the robustness of the fitting method to variation in sample size by varying the size of the random samples drawn from each original distribution, i.e., by simulating different numbers of retrieved propagules (ranging from N = 50 to N = 1500). We restricted this analysis to a single type of original distribution, and chose to use the lognormal distribution due to its wide use in studies describing gut retention time in different animal vectors [4, 33]. The lognormal distribution was defined with parameter values corresponding to the mean of the parameters’ range reported in Viana et al. [4]. Sampled data for each simulation were assigned to sampling time-intervals corresponding to the lengths that provided the best performance (see Results; below we explain interval length variation).

#### Variation in the length of sampling time-intervals

We assessed the robustness of the fitting method to different sampling time-intervals by varying, either uniformly or asymmetrically, the length of the time intervals used to sample data from the original distribution. We used both regular intervals over the whole sampling period, using three different lengths (either 1, 2 or 4 h, throughout the whole range of 52 h), and variable interval lengths. The latter varied in length (i) around the distribution mode, defining intervals of 1, 2 or 4 h during the first 8 h followed in all three cases by intervals of 4 h throughout the remaining sampling period, (ii) in the tail of the distribution, defining intervals of 1 h up to 8 h followed by intervals of either 2, 4 or 8 h afterwards, or (iii) only in the last interval, defining intervals of 1 h until 8 h and 4 h until either 12, 24 or 36 h followed by a variable last sampling bout at the end of the sampling procedure (52 h). All these sampling schemes reproduce procedures used in published studies [e.g., 19, 24, 27, 37], though additional variation was introduced in some of them. We used the lognormal distribution as the original distribution and a sample size of 500 propagules.

#### Impact of fitting method on estimates of propagule dispersal kernels

To assess the bias produced by non-optimal fitting methods on various dispersal kernel properties, we compared the discrepancy in four dispersal kernel parameters (long-distance dispersal frequency, mean and median distance, and the 99th distance percentile) estimated using retention-time distributions fitted according to the five fitting methods described in the previous sections (only lognormal distributions were used). For this purpose, we used two examples [from 24, 37] in which the seed retention times of two aquatic plant species (*P. pectinatus* and *S. lacustris*) in the guts of a single waterfowl species (mallard *Anas platyrhynchos*) were measured using different sampling time-intervals (every 4 h for *P. pectinatusversus* every hour up to 4 h, every 2 h up to 8 h, and every 4 h afterwards for *S. lacustris*). Dispersal kernels were estimated using a realistic distribution of vector movement distances, based on waterfowl banding data (data available in [4]). For each of the four dispersal kernel parameters, we calculated the relative difference (in percentage) between the value obtained using the optimal method (CD-ML; see results) and each of all other fitting methods.

## Availability of data and materials

The datasets supporting the conclusions of this article are available in the Dryad Digital Repository, http://datadryad.org/resource/doi:10.5061/dryad.619gd.
